# Serum gamma-glutamyltransferase and the overall survival of metastatic pancreatic cancer

**DOI:** 10.1186/s12885-019-6250-8

**Published:** 2019-10-29

**Authors:** Yuanyuan Xiao, Haijun Yang, Jian Lu, Dehui Li, Chuanzhi Xu, Harvey A. Risch

**Affiliations:** 1 School of Public Health, Kunming Medical University, 1168 West Chunrong Road, Kunming, Yunnan China; 2 Department of Chronic Disease Epidemiology, Yale School of Public Health, Yale University, New Haven, CT 06520-8034 USA; 3 The Third Affiliated Hospital of Kunming Medical University, Kunming, Yunnan China

**Keywords:** Biomarkers, Pancreatic cancer, Survival

## Abstract

**Background:**

Accumulating evidence suggests that Gamma-glutamyltransferase (GGT) may be involved in cancer occurrence and progression. However, the prognostic role of serum GGT in pancreatic cancer (PC) survival lacks adequate evaluation. In this study, we aimed to analyze the association between serum GGT measured at diagnosis and overall survival (OS) in patients with metastatic PC.

**Methods:**

We identified 320 patients with histopathologically confirmed metastatic pancreatic ductal adenocarcinoma (PDAC) diagnosed during 2015 and 2016 at a specialized cancer hospital in southwestern China. Univariate and multivariate Cox proportional-hazards models were used to determine associations between serum GGT and OS in metastatic PDAC.

**Results:**

Controlled for possible confounding factors, serum GGT was significantly associated with OS: serum GGT > 48 U/L yielded a hazard ratio of 1.53 (95% CI: 1.19–1.97) for mortality risk. A significant dose-response association between serum GGT and OS was also observed. Subgroup analysis showed a possible interaction between GGT and blood glucose level.

**Conclusion:**

Serum GGT could be a potential indicator of survival in metastatic PDAC patients. Underlying mechanisms for this association should be investigated.

## Background

The enzyme gamma-glutamyltransferase (GGT) plays a key role in glutathione metabolism. Located primarily on the plasma membrane of the cell, it passes amino acids across the cell membrane and transfers glutamyl onto water, peptides and other recipient molecules. Although GGT is nearly omnipresent in human tissues [1], circulating GGT is thought to be derived predominantly from the liver [2]. Therefore, in clinical practice, blood levels are widely used as a sensitive albeit nonspecific diagnostic biomarker for liver dysfunction, hepatitis and excessive alcohol consumption. In the past two decades, elevated GGT levels have also been associated with increased risk of various chronic diseases, such as cardiovascular events [3], hypertension [4], type II diabetes [5], metabolic syndrome [6], and renal failure [7].

Pro-oxidation and pro-inflammatory properties of GGT have been suggested to underlie etiological mechanisms leading to these adverse disease outcomes [8]. Oxidative stress and inflammation are also pathways implicated in cancer development and progression, and positive GGT associations with risks of various cancer types have been demonstrated by several prospective studies [9–12]. Quite opposite to the abundance of cancer risk studies, relatively few studies have investigated the prognostic role of serum GGT in cancer outcomes: increased serum GGT has been inversely associated with survival in gastric cancer [13], colorectal cancer [14], ovarian cancer [15], breast cancer [16], endometrial cancer [17, 18], cervical cancer [19], and renal cell carcinoma [20].

Pancreatic cancer (PC) remains one of the most lethal malignant tumors worldwide, with a dismal 5-year survival proportion of less than 5% [21]. Thus, identification of factors that are of prognostic significance is imperative. In parallel to the aforementioned inverse survival associations with other malignancies, it is possible that serum GGT may also relate to PC survival. Two studies have examined the relations of serum GGT and GGT-to-albumin ratio with PC survival in early stage patients, with positive findings [22, 23]. Few studies have investigated the prognostic relevance of serum GGT in advanced PC patients [24]. Therefore, the aim of this study was to assess the association between serum GGT measured at disease diagnosis and overall survival (OS) in PC patients with metastatic disease.

## Methods

### Study design

After the Institutional Research Ethics Board of Kunming Medical University reviewed and approved the protocol, we performed a retrospective study at The Third Affiliated Hospital of Kunming University, the largest cancer hospital in southwestern China Yunnan province. This hospital has a comprehensive computerized information system that collects, verifies and updates all parts of medical practice-relevant data from inpatients and outpatients on a daily basis, including hospitalization records, imaging examinations, body-fluids tests, drug prescriptions, diagnoses, surgical procedures, etc. In this study, we screened the database for histopathologically confirmed stage IV (metastatic) pancreatic ductal adenocarcinoma (PDAC) patients diagnosed between January 1, 2015 and December 31, 2016. Other information that was obtained from the system included age at diagnosis, sex, blood indicators and chemotherapy data. Information about deaths of the included patients through January 1, 2018 was ascertained by external matching with the Chinese Residents Death Registration System, using the individual personal ID number assigned to every Chinese citizen. Because of the retrospective nature of the study, informed consents from the subjects were waived by the institutional review board.

### Variables and definitions

The outcome of interest in this study was OS. Survival duration was defined as the time between histopathological diagnosis date and death date. Data on baseline serum GGT level for each included patient, measured within 7 days of diagnosis, were extracted from the information system. Considering that nutritional status, liver function, hyperglycemia and systemic inflammation may introduce confounding in analyses of the association between serum GGT and OS, we also extracted baseline test results of several other blood indicators: albumin (ALB), an indicator of nutritional status; total bilirubin (TBIL) and alanine transaminase (ALT), measurements of liver function; fasting plasma glucose (FPG), a marker of blood glucose level; and neutrophil-to-lymphocyte ratio (NLR), a measure of systemic inflammation. Chemotherapy of the patients was with palliative intention, and was defined as the single or combination use of any of the following commonly used drugs: gemcitabine, nab-paclitaxel, 5-fluorouracil, irinotecan, and oxaliplatin.

### Statistical analysis

Descriptive statistics were used to delineate and compare general characteristics of the study subjects. Univariate and multivariate Cox proportional-hazards models were applied to evaluate the association between baseline serum GGT level and PDAC OS. Subgroup analyses based on chemotherapy, NLR values, and blood glucose levels were performed to examine possible GGT interaction roles. All statistical analyses were done in SAS (version 9.3, SAS Institute Inc., Cary, NC.). Except for the exploratory univariate Cox models, which employed a less strict standard (*p* < 0.10) for initial identification of possible influencing factors, the threshold for nominal statistical significance was defined as a two-tailed probability less than 0.05.

## Results

### General characteristics of PDAC patients

Over the 2 years of subject eligibility, we identified 357 patients with histologically confirmed metastatic PDAC, of whom 37 had missing values in critical variables. Thus, our analyses included 320 patients. Major characteristics of the patients are described and compared in Table 1. The mean diagnosis age of patients was 65.3 years, males and females comparable. Median survival was 177 days. Although serum GGT can mildly vary by age and sex, in clinical practice, a uniform cut-off of 48 units/liter (U/L) is the most commonly used threshold for defining GGT elevation. We chose this value a priori to dichotomize the PDAC patients based on baseline serum GGT level. We found that, except for age, sex and serum FPG, the various ascertained characteristics were all significantly different between the two groups: compared to patients with normal baseline serum GGT, patients with elevated levels had much shorter median survival (138 versus 281 days), as well as generally higher other blood markers.

**Table 1:** General characteristics of 320 metastatic PDAC patients

Characteristics	All patients (*N* = 320)	Elevated serum GGT (GGT > 48 U/L, *N* = 183)	Normal serum GGT (GGT ≤ 48 U/L, *N* = 137)	*p* value
	Mean (SD)/Median (SD)/*N* (%)	Mean (SD)/Median (SD)/*N* (%)	Mean (SD)/Median (SD)/*N* (%)	
Age at diagnosis (Years)	65.28 (10.05) ^a^	65.93 (9.97) ^a^	64.41 (10.14) ^a^	0.18
Sex (Male)	158 (49.38) ^c^	93 (50.82) ^c^	65 (47.45) ^c^	0.57
Palliative chemotherapy (Yes)	150 (46.88) ^c^	63 (34.43) ^c^	87 (63.50) ^c^	10^–6.5^
Survival length (Days)	177 (231.93) ^b^	138 (271.65) ^b^	281 (234.19) ^b^	10^–6.0^
Baseline serum indicators				
ALB (g/L)	37.00 (7.32) ^b^	36.00 (6.96) ^b^	39.30 (7.12) ^b^	10^–6.5^
TBIL (μmol/L)	15.40 (113.14) ^b^	34.80 (137.81) ^b^	11.20 (15.36) ^b^	10^−14^
ALT (U/L)	29.00 (107.87) ^b^	58.00 (110.61) ^b^	17.10 (91.23) ^b^	10^–8.1^
FPG (mmol/L)	6.67 (3.26) ^b^	6.70 (3.78) ^b^	6.42 (2.32) ^b^	0.15
NLR (Unit free)	4.46 (8.26) ^b^	5.08 (8.13) ^b^	3.74 (8.45) ^b^	10^–2.3^
GGT (U/L)	85.5 (393.02) ^b^	NA	NA	NA

^a^ Mean with standard deviation (SD)

^b^ Median with standard deviation (SD)

^c^ Frequency with proportion (%)

### Baseline serum GGT and OS of metastatic PDAC

**Fig. 1:**
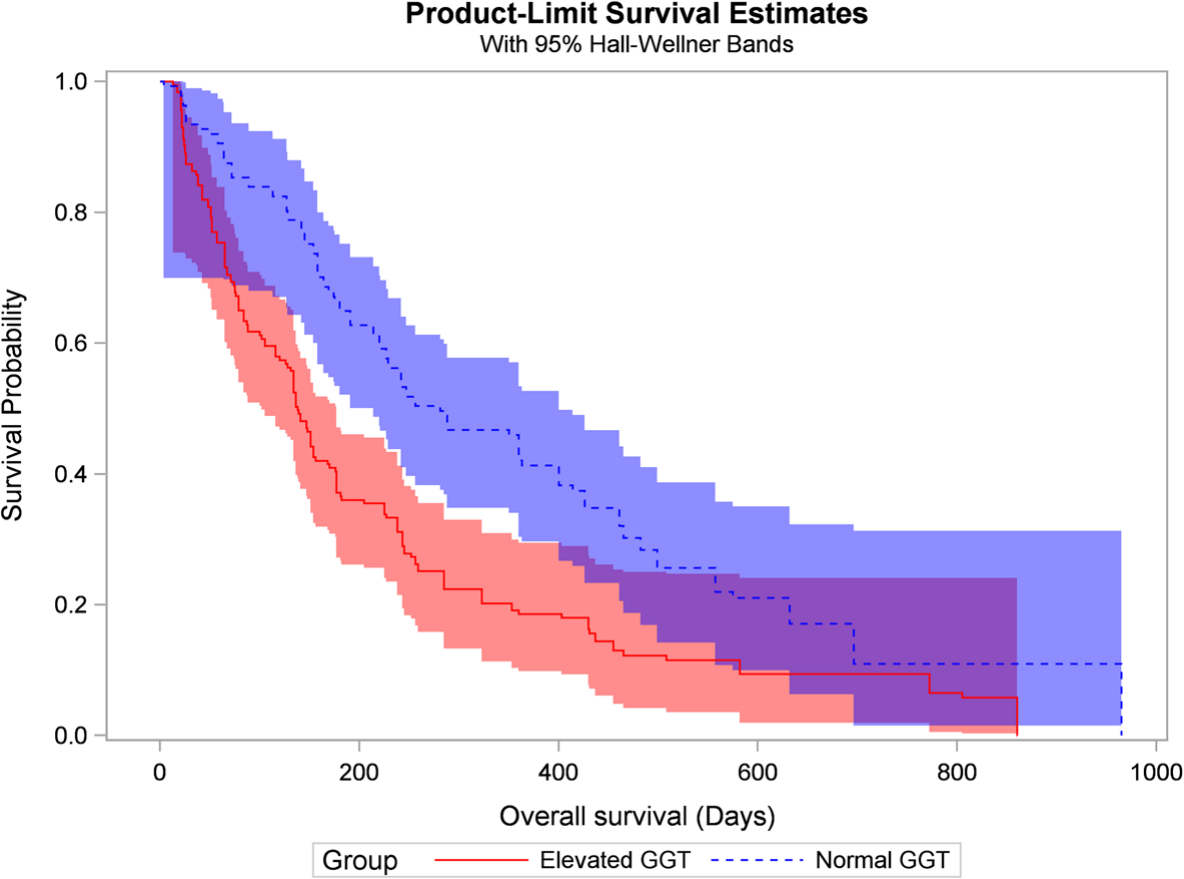
Kaplan-Meier survival curves for metastatic PDAC patients with elevated and normal baseline serum GGT levels. Log-rank statistic χ^2^ = 23.5, *p* = 10^− 6^. PDAC, pancreatic ductal adenocarcinoma; GGT, Gamma-glutamyltransferase

Product-limit survival curves of elevated and normal baseline serum GGT patients are displayed in Fig. 1. OS of the elevated GGT group was notably inferior to survival of the normal GGT group (log-rank statistic: 23.52, *p* = 10^− 6^). Univariate Cox proportional-hazards models identified 4 potential prognostic covariates: age at diagnosis, palliative chemotherapy, baseline FPG and baseline GGT. With multivariate adjustment, only age at diagnosis, FPG and GGT remained significant. Age at diagnosis was positively associated with mortality: the adjusted hazard ratio (HR) was 1.08 (95% CI 1.01–1.15) per 5 years increase; elevated baseline FPG and serum GGT were associated with 1.39- (95% CI: 1.08–1.79) and 1.53- (95% CI: 1.19–1.97) fold mortality, respectively (Table 2).

**Table 2:** Univariate and multivariate Cox proportional hazards model results

Covariates	Univariate Cox model		Multivariate Cox model	
	Crude HR (95% CI)	*p* value	Adjusted HR (95% CI)	*p* value
Age at diagnosis (+ 5 years)	1.10 (1.03, 1.16)	10^–2.9^	1.08 (1.01, 1.15)	10^–1.7^
Sex (Male)	1.02 (0.81, 1.29)	0.87		
Palliative chemotherapy (Yes)	0.69 (0.54, 0.87)	10^–2.9^	0.81 (0.62, 1.04)	0.10
Baseline serum ALB (< 35 g/L)	1.25 (0.98, 1.59)	0.10		
Baseline serum TBIL (> 20.5 μmol/L)	1.19 (0.93, 1.50)	0.16		
Baseline serum ALT (> 60 U/L)	1.17 (0.91, 1.50)	0.22		
Baseline serum NLR (+ 10)	1.10 (0.97, 1.25)	0.15		
Baseline FPG (≥7.0 mmol/L)	1.32 (1.04, 1.68)	10^–1.7^	1.39 (1.08, 1.79)	10^–2.0^
Baseline serum GGT (> 48 U/L)	1.78 (1.40, 2.26)	10^–6.0^	1.53 (1.19, 1.97)	10^–3.2^

**Fig. 2:**
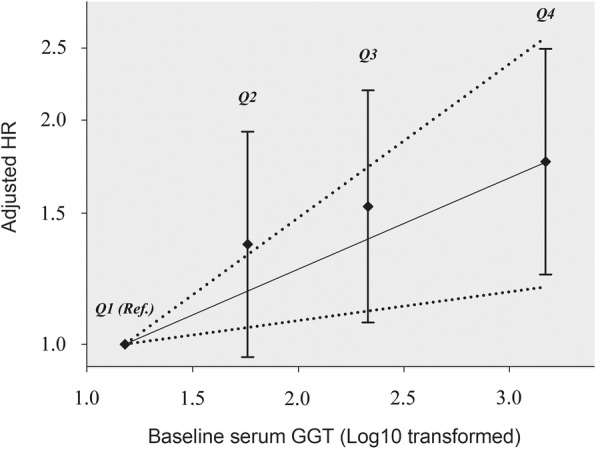
Dose-response association between baseline serum GGT and the OS in metastatic PDAC patients. Adjusted for age at diagnosis, palliative chemotherapy, baseline ALB, and baseline FPG. The estimated dose-response trend and its 95% confidence band are given in solid and dotted lines, respectively. The Y-axis represents 1og_10_(HR), thus intervals are not equally spaced. GGT, Gamma-glutamyltransferase; ALB, albumin; FPG, fasting plasma glucose

We divided PDAC patients into 4 strata by quartile of baseline serum GGT: Q_1_ (GGT < 30.0 U/L), Q_2_ (30.0 U/L ≤ GGT < 85.5 U/L), Q_3_ (85.5 U/L ≤ GGT < 338.0 U/L), and Q_4_ (GGT ≥ 338.0 U/L). By using Q_1_ as the reference group, controlling for age at diagnosis, palliative chemotherapy, baseline FPG and baseline ALB, we found that the adjusted HRs for Q_2_ through Q_4_ were 1.36 (95% CI: 0.96–1.93), 1.53 (95% CI: 1.07–2.19), and 1.76 (95% CI: 1.24–2.49), respectively. The multiplicative continuous dose-response association between GGT and OS was statistically significant: every 10-fold increase in GGT was associated with a HR of 1.33 (95% CI: 1.09–1.61), and the *p* value for this continuous trend was 0.0043 (Fig. 2).

### Subgroup analysis

We further performed a small series of subgroup analyses based on GGT stratification by categories of palliative chemotherapy, baseline FPG and NLR. No obvious interaction was found between palliative chemotherapy, baseline NLR and serum GGT. However, an appreciable difference in the GGT-OS association was found when metastatic PDAC patients were dichotomized by baseline FPG: in patients with elevated baseline FPG (defined as ≥7.0 mmol/L), GGT was not associated with OS, but in patients with normal baseline FPG (defined as < 7.0 mmol/L), elevated serum GGT was associated with 2.14-fold mortality (95% CI: 1.48–3.09) (Table 3). This interaction did not reach statistical significance however (*p* = 0.07).

**Table 3:** Subgroup analysis results by chemotherapy, baseline FPG and NLR

Stratification variable	Stratum	Elevated serum GGT (> 48 U/L)	
		Adjusted HR (95% CI)	Interaction *p*-value
Palliative chemotherapy	Yes	1.70 (1.16, 2.51) ^a^	0.84
	No	1.61 (1.10, 2.34) ^a^	
Baseline NLR	≥ 4.46	1.38 (0.95, 2.00) ^b^	0.91
	< 4.46	1.42 (0.99, 2.03) ^b^	
Baseline FPG	≥ 7.0 mmol/L	1.31 (0.90, 1.92) ^c^	0.07
	< 7.0 mmol/L	2.14 (1.48, 3.09) ^c^	

^a^ Adjusted for age at diagnosis, baseline NLR, baseline FPG

^b^ Adjusted for age at diagnosis, palliative chemotherapy, baseline FPG

^c^ Adjusted for age at diagnosis, palliative chemotherapy, baseline NLR

Among the 320 PDAC patients, 76 and 97 were additionally measured for baseline C-reactive protein (CRP) and carbohydrate antigen 19–9 (CA19–9), respectively. Correlation analysis revealed that serum GGT was positively associated with CA19–9 (*r* = 0.43, *p* = 10^–3.9^), whereas the relationship between GGT and CRP was negligible (*r* = − 0.01, *p* = 0.97). Considering the appreciable correlation between GGT and CA19–9, we fitted a multivariate Cox regression model that included both CA19–9 and GGT in the subset of the 97 PDAC patients with baseline CA19–9 measurements: elevated GGT was still a significant prognostic factor (HR = 1.53, 95% CI: 1.10–2.13), and CA19–9 was not significantly associated with OS (HR = 1.50, 95% CI: 0.78–2.85).

## Discussion

In this study, we examined the prognostic relevance of serum GGT measured at diagnosis among histopathologically diagnosed metastatic PDAC patients. We found that baseline serum GGT was positively associated with OS: compared to patients with normal GGT, elevated GGT showed a 54% increase in mortality hazard. A previous study by Engelken et al. also reported a significant association between elevated GGT and deteriorated survival in unresectable PDAC patients. However, those authors adopted an unusual 3.4-fold higher cut-off (165 U/L) to dichotomize GGT level [24]. Our findings were further supported by an appreciable dose-response trend of increasing mortality hazard with increasing GGT level. This association is of potential clinical significance: as a prognostic factor. A possible role of serum GGT in cancer survival should be investigated, in the hope of elucidating modifiable survival mechanisms in these cancer patients.

Laboratory studies the involvement of serum GGT in metastatic PDAC prognosis. First, evidence supports a role of inflammation in cancer progression. For example, nuclear factor-κB (NF-κB) is involved in inflammation-induced tumor growth [25]. The association of GGT with inflammation has been observed bidirectionally: elevated serum GGT can reflect inflammation-related oxidative stress [26], or can be a consequence of inflammatory cytokines such as tumor necrosis factor alpha [20]. However, our observed mortality association with serum GGT was not affected by adjustment for baseline NLR, a sensitive marker of systemic inflammation, raising the possibility that mechanisms other than inflammation may also exist in this association. For example, GGT may directly participate in the progression of cancer, as studies on melanoma cells found that elevated GGT activity resulted in a growth advantage both in vitro and in vivo [27]. Moreover, a newly published study using gene-set enrichment analysis (GSEA) reported that in gastric cancer patients, GGT was significantly associated with EMT, KRAS, SRC and PKCA signaling pathways [13], which are involved in cancer progression and metastasis [28, 29].

The connection between hyperglycemia and cancer progression has been well established [30]. Our study results are consistent with this association, as we observed that elevated baseline fasting glucose level was associated with increased mortality hazard. However, subgroup analysis in strata of FPG suggested that serum GGT was significantly associated with increased mortality hazard primarily in patients with normal blood glucose levels rather than in their hyperglycemic counterparts. A reasonable hypothesis could be that normal rather than increased plasma glucose level may enhance the bioactivity of serum GGT. However, we cannot find direct laboratory evidence to support this mechanism. Two previous studies have examined the association between serum GGT and glucose levels: one reported a positive association [31], whereas the other reached a nonsignificant conclusion [32]. Another possible explanation could be a pro-inflammatory effect of hypoglycemia that has been noted recently [33, 34], as induced inflammation may exacerbate of the association with elevated GGT. Nevertheless, it is also understood that hyperglycemia has a similar pro-inflammation propensity [35, 36]. Therefore, this issue needs to be further explored.

If validated, the findings of this study may have clinical significance. As an identified prognostic factor, serum GGT level in metastatic PDAC patients could be periodically monitored. As an accompanying indicator of disease progression, serum GGT could help to predict more imminent mortality. On the other hand, if serum GGT independently promotes disease progression, then reducing its level could be a potential strategy in improving survival. Although currently only toxic GGT inhibitors are available [37, 38], low toxicity analogs that can be used in humans are in the process of development.

The strength of our study is the comparatively large sample size for this uncommon disease. Nevertheless, several limitations should also be noted. First, all of our study subjects were metastatic PDAC patients chosen from a single Chinese institution, thus generalization of the study results to PC patients with other disease stages or of other ethnic backgrounds should be made cautiously. Second, although we included some potentially important factors for adjustment of the study results, other potential confounders for which we had no information, such as tumor location, smoking history, and alcohol consumption, were not adjusted. Therefore, some unadjusted confounding biases could still exist.

## Conclusions

Increased baseline GGT was associated with lower OS of metastatic PDAC patients. Some evidence for mortality interaction was observed between blood glucose level and serum GGT. Our study results suggest that serum GGT might be used as an indicator to identify late stage PDAC patients with increased mortality hazard. These findings warrant corroboration by studies with larger sample sizes, and mechanisms to explain this association also need to be investigated.

## Data Availability

The datasets analyzed in the current study are not publicly available due to confidentiality agreements, but are available from the corresponding author subject to approval by the Institutional Research Ethics Board of Kunming University.

## Abbreviations

ALB: Albumin
ALT: Alanine transaminase
FPG: Fasting plasma glucose
GGT: Gamma-glutamyltransferase (GGT)
HR: Hazard ratio
NLR: Neutrophil-to-lymphocyte ratio
PDAC: Pancreatic adenocarcinoma
TBIL: Total bilirubin
